# Highlighting the Role of DCLK1 in Tumor Invasion and Potential for Therapeutic Intervention

**DOI:** 10.33696/cancerimmunol.7.116

**Published:** 2025

**Authors:** Molly E. Muehlebach, Sufi Mary Thomas

**Affiliations:** 1 Department of Otolaryngology – Head and Neck Surgery, University of Kansas Medical Center, Kansas City, KS, USA

**Keywords:** Head and neck squamous cell carcinoma, Squamous cell carcinomas, Tumors

## Abstract

Head and neck squamous cell carcinoma (HNSCC) accounts for nearly 5% of global cancer deaths per year, with epidemiological studies suggesting an expected 30% increase in cases by 2030 due to rising incidence of viral infection (i.e. huma papilloma virus [HPV]). Treatment consists primarily of surgical tumor removal accompanied by post-operative chemoradiation therapy; however, disease recurrence is still an issue amongst 10–26% of patients. Doublecortin-like kinase 1 (DCLK1) is a microtubule-associated protein with dual kinase activity, and upregulation has been associated with poor prognosis in multiple solid tumors. Recent studies by Arnold *et al.* highlighted a definitive role of DCLK1 in HNSCC invadopodium formation and function, and has elucidated its potential for small molecule targeting. This commentary summarizes the molecular findings by Arnold *et al.* and evaluates them in regard to the current standings of DCLK1 targeting, emphasizing why inhibiting this molecule could be of clinical significance.

## Commentary

Head and neck cancer is the seventh most common malignancy worldwide with majority (~90%) classified as squamous cell carcinomas (HNSCC) [1,2]. HNSCC arises from the mucosal tissues of the oral cavity and pharynx due to factors such as genetic predisposition, tobacco and/or alcohol use, diet, and/or viral infection (human papilloma virus (HPV)) to name a few [1,3]. Treatment regimens primarily consist of surgical removal followed by postoperative chemoradiation however, disease recurrence remains an issue amongst 10–26% of patients [3-8]. Due to the anatomical location of HNSCC, disease recurrence and locoregional dissemination is a major risk factor, with lymphovascular and perineural invasion contributing to mortality [9].

Epidermal growth factor receptor (EGFR) signaling is recognized as a primary mediator of HNSCC invasion as EGFR expression is known to be elevated particularly in HPV negative disease [10,11]. HNSCC EGFR activation has also been shown to directly regulate formation of cancer cell invasive machinery termed invadopodia [10,12]. Invadopodia are dynamic, actin-rich protrusions which mediate extracellular matrix (ECM) proteolysis allowing for HNSCC migration and invasion into external structures [13]. Essentially transformed podosomes, these “invasive feet” are considered unique to cancer cells highlighting their potential as therapeutic targets [14]. EGFR inhibition has been shown to impair HNSCC invasion, leading to the approval of EGFR monoclonal antibody cetuximab as a therapeutic for these patients [15,16]. However, better understanding of the downstream molecular components which mediate invadopodia formation remains an area of investigation.

Invadopodia formation occurs on the ventral cell membrane usually in response to different growth factors (i.e. EGF, FGF, VEGF, PDGF) but can also occur in response to integrin-related extracellular signals, and/or environmental changes (i.e. acidic pH, matrix rigidity, hypoxia, ROS) [17,18]. These signals trigger what is referred to as the initiation phase, in which activated receptors stimulate signaling cascades primarily mediated by Src activation and phosphorylation of downstream targets such as tyrosine kinase substrate with five SH3 domains (TKS5) [14,18]. TKS5 can then activate actin regulator cortactin, known to be overexpressed in HNSCC cells, allowing for release of actin capping protein cofilin for stimulation of actin polymerization [19,20]. With the assembly of other actin regulators such as ARP2/3, N-WASP, and WIP, adaptor proteins such as TKS4 coordinate trafficking of matrix metalloproteases (MMP) −9, −2, and −14, to the invadopodium leading edge for focal ECM degradation [20-22]. While EGFR inhibition has been deemed efficacious in targeting invasion in these patients, further investigation into targeting direct invadopodia machinery is of interest.

Doublecortin-like kinase 1 (DCLK1) is a bi-functional protein with dual microtubule-associated properties (MAP) and serine/threonine kinase function, shown to be overexpressed in cancer. Early investigation into DCLK1 in normal physiology revealed a regulatory role in early neurogenesis as well as mediating neuronal migration and microtubule polymerization [23-25]. However, in recent years, DCLK1 has been implicated as a cancer stem cell marker for gastrointestinal, colorectal, and pancreatic malignancies with higher expression levels correlating with advanced disease stage [26-31]. Furthermore, expression has been associated with poor prognosis in breast cancer (BC), prostate cancer, bladder cancer, and renal cell carcinoma (RCC), indicating DCLK1 significance in cancer development and progression [32,33]. In the case of colorectal cancer (CRC) and pancreatic ductal adenocarcinoma (PDAC), DCLK1 may serve as a marker for tumor initiating cells with DCLK1-positive cells highly upregulated in neoplastic lesions [33]. However, pro-tumorigenic properties in general are majorly attributed to activation of the epithelial-to-mesenchymal transition (EMT) via kinase-mediated phosphorylation of ERK signaling [33]. DCLK1-mediated ERK activation has been found to downregulate E-cadherin and increase expression of mesenchymal markers such as ZEB-1, SLUG, vimentin, and SNAIL in aggregate promoting tumor invasion, metastasis, and therapeutic resistance [34].

Importantly, human DCLK1 includes both long (DCLK1-L) and short (DCLK1-S) isoforms with each being associated with unique biological activity and transcriptionally regulated by distinct promoter regions [32]. The DCLK1-S isoform, lacking the doublecortin (DCX) microtubule-binding domains, is shown to be preferentially upregulated in CRC [32]. Alternatively, the long isoform has been associated with RCC while both short and long isoforms having been detected in PDAC [32]. Therefore, expression of not just DCLK1 but the specific isoform in solid cancers may impact pro-tumorigenic effects.

Heightened DCLK1 expression (isoform not specified) has also been confirmed in HNSCC cohorts with high TCGA mRNA expression data correlating with reduced survival in these patients [35]. Specifically, high expression is correlated with HPV negative disease which is known to be more aggressive than HPV positive HNSCC and associated with poor response to therapy [35]. Elevated expression was correlated with higher lymph node stage and histological tumor grade, indicating a role in disease metastasis [36]. With this data, Arnold *et al.* tested a hypothesis that implicates a molecular role of DCLK1 in HNSCC tumor cell invasion and elucidated its potential as a therapeutic target [36].

Initial immunohistochemical analysis of HNSCC patients samples highlighted a distinct localization of DCLK1 to the leading edge of tumors [36]. To confirm whether or not DCLK1 was truly implicated in HNSCC invasive potential, shRNA knockdown was assessed and reduced HNSCC cell migration was confirmed [35,36]. To take an unbiased approach, control and DCLK1 shRNA HNSCC cells were analyzed with tandem proteomic and phosphoproteomic analysis [36]. Protein expression was significantly altered in shDCLK1 cells, with ontological analysis revealing association of DCLK1-enriched proteins with ECM interaction, cytoskeletal reorganization, and cell adhesion [36]. Furthermore, specific associations between DCLK1 and kinases associated with cell locomotion and cytoskeletal rearrangement (EGFR, ERK1/2, Src, and PAK1) were lost in shDCLK1 cells [36]. Use of immunofluorescent microscopy and gelatin invadopodia assays, confirmed DCLK1 localization with actin in areas of high gelatin degradation [36]. Alternatively, DCLK1 knockdown cells exhibited reduced number of cellular projections and importantly, use of DCLK1 specific inhibitor, 3,5-bis (2,4-difluorobenzylidene)-4-piperidone (DiFiD), significantly reduced gelatin degradation [36]. In cumulative, DCLK1 knockdown, impaired HNSCC cell invasion and matrix degradation, with confirmed alterations in phosphorylation of key signaling mediators for invadopodia initiation and formation (EGFR, ERK1/2, Src, PAK1).

**Figure 1.**
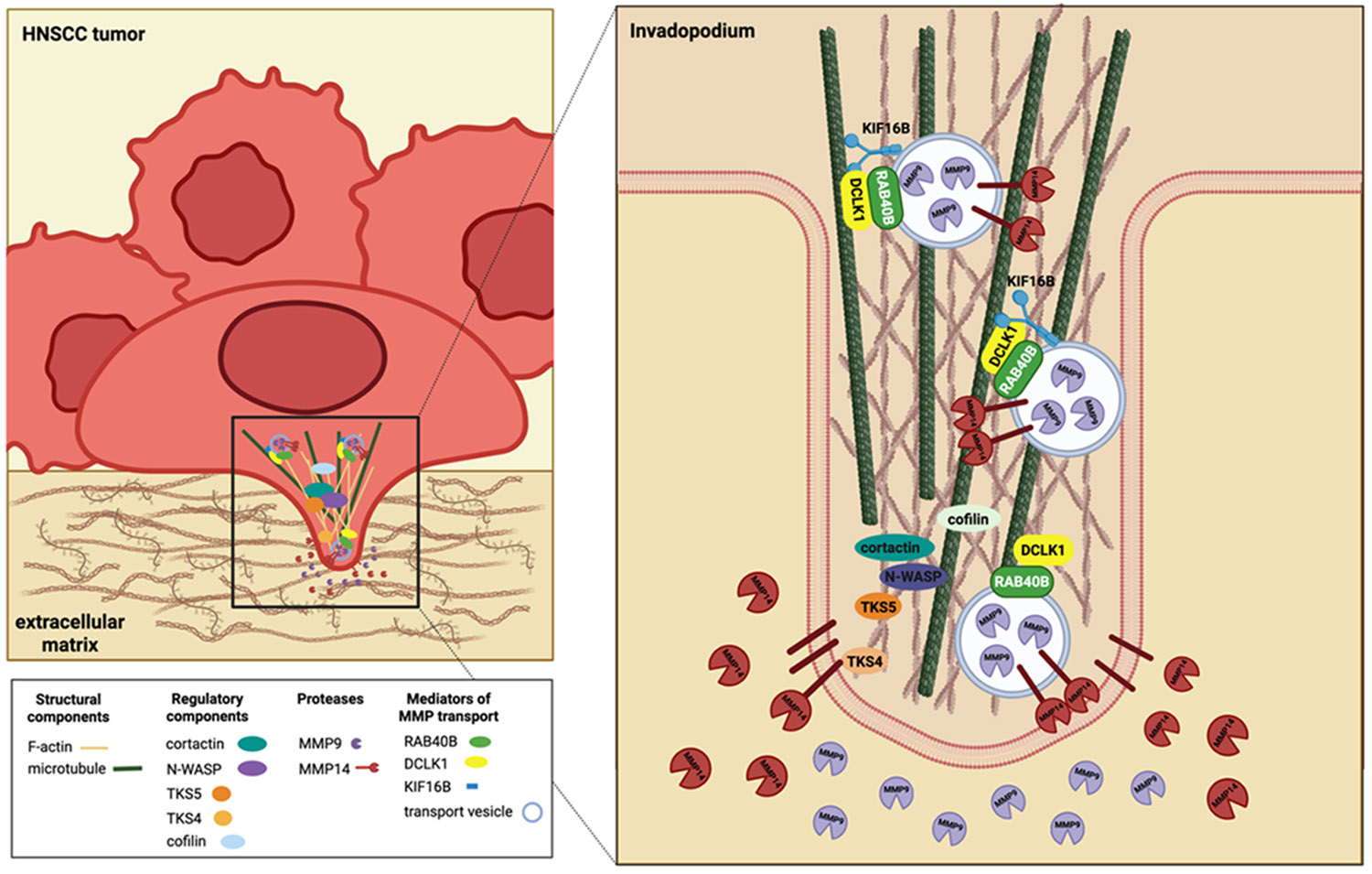
DCLK1 mediates microtubule trafficking of MMPs to the invadopodium leading edge for extracellular matrix degradation.

Immunofluorescent colocalization studies confirmed DCLK1 to localize to invadopodia of HNSCC cells with proximity to mature invadopodia machinery such as TKS4, TKS5, cortactin, and MMP14 [36]. Furthermore, colocalization with these proteins was reduced as distance from the invadopodia leading edge increased [36]. MMP array and zymography studies highlighted MMP9, MMP13, and MMP1 to be reduced in shDCLK1 cells, confirming DCLK1 to play a role not only in early invadopodia formation, but also functional mediation of matrix degradation [36]. Considering DCLK1’s microtubule-associated function and role in microtubule transport in neurobiology, DCLK1 was hypothesized to mediate MMP transport to the invadopodia leading edge. A pan-kinesin scan of TCGA data revealed DCLK1 expression correlated with the kinesin 3 subfamily of motor proteins across tumor stages, with proximity ligation assays and co-immunoprecipitation confirming motor protein, KIF16B, to interact with DCLK1 [36]. With immunofluorescent microscopy, DCLK1 was confirmed to interact with KIF16B and intracellular transport protein RAB40B to mediate MMP transport [36]. Furthermore, Phosphositeplus confirmed potential DCLK1 phosphorylation sites on the C-terminal tail of RAB40B and residues in the motor domain of KIF16B. Considering that RAB40B is commonly associated with the transport of MMP9 and MMP14, Arnold *et al.* concluded that DCLK1 facilitates microtubule transport of MMP enzymes such as MMP9 and MMP14, in order to degrade resident matrix and allow for intravasation of surrounding extracellular matrix (Figure 1) [36].

These studies were the first to highlight a regulatory role of DCLK1 in invadopodia formation and degradation. However, previous research in dendrites have shown DCLK1 to mediate kinesin-3-dependent cargo transport along microtubules [37]. In these studies, the microtubule binding domain of DCLK1 was found to directly interact with kinesin motor domains. It was hypothesized that the DCX domain may mediate KIF16B detachment from microtubules after completion of its ATPase cycle. However, further studies are needed to confirm this interaction. Furthermore, other DCX microtubule associated functions include DCLK1 binding of tubulin protofilaments, which could also explain the colocalization witnessed in the HNSCC studies [38]. Therefore, suggesting the need for continued investigation into the direct interaction of DCLK1 with motor protein KIF16B.

While these studies are the first to confirm a direct regulatory role for DCLK1 in cell invasion machinery, recent data in esophageal squamous cell carcinoma (ESCC) and metastatic BC further supports the role of DCLK1 in MMP regulation and invasion [39,40]. Upregulation of DCLK1-S has been associated with malignant progression and poor prognosis in ESCC [39]. *In vitro* studies confirmed DCLK1-knockout in ESCC cell lines to reduce colony formation and invasion, also replicated by inhibition with DCLK1 kinase inhibitor LRRK2-IN-1 [39]. Injection of DCLK1-S expressing cells in mice were shown to accelerate tumor growth and metastatic lung colonization with these pro-tumorigenic properties associated with ERK1/2 pathway activation inducing MMP2 expression for EMT [39]. This was confirmed by use of ERK1/2 antagonist, SCH772984, inhibiting DCLK1-S-mediated ESCC proliferation and migration [39]. While MMP2 was not recognized as one of the major MMPs regulated by DCLK1 in the study by Arnold *et al.*, phosphoproteomic data did indicate a positive regulatory role of DCLK1 on ERK signaling. Furthermore, DCLK1 overexpression in BC cell lines was found to markedly increase cell migration and invasion, with these processes being inhibited with DCLK1 knockout [40]. Evaluation of ERK activation revealed knockout of DCLK1 to inhibit phosphorylation of ERK, downregulating expression of MMP14 [40]. These data are in agreement with that from Arnold *et al.*, suggesting MMP14 regulation in HNSCC cells may be ERK-specific.

Throughout the Arnold *et al.* studies, DCLK1 inhibitor, DiFiD, was repeatedly used to show loss of invadopodia formation and matrix degrading ability with kinase inhibition [35]. Previous molecular docking studies predicted DiFiD to form hydrogen bonds with aspartic acid 533 within the kinase domain, but whether binding impacts MAP function needs clarification [35]. *In vitro* kinase assays assessing the effects of DiFiD on closely related CaM kinase members, has confirmed specificity for DCLK1, indicating potential for minimal off-target effects with this compound [35]. This was further confirmed by lack of DiFiD cytotoxic effects in the Het1A non-cancerous cell line and DCLK1 knockdown cells [35]. DiFiD has previously been investigated in HNSCC cells where it was proven to inhibit colony formation and induce G2/M arrest [35]. DiFiD has also exhibited antineoplastic effects *in vivo*, significantly reducing HNSCC tumor growth [35]. Importantly, the compound was thought to be well tolerated due to no significant changes in body weight [35]. This finding is significant considering that healthy neurons and tuft cells found in the colon express DCLK1. While this suggests minimal toxicity in normal tissues, future studies should evaluate specific neuronal and intestinal changes to determine whether on-target effects impact function of these organ systems. While further investigation of the compound is needed *in vivo*, the use of DiFiD as a molecular tool has elucidated the role of DCLK1 in HNSCC malignancy and indicated its potential for targeted therapy.

Other small molecule inhibitors of DCLK1 have been investigated over the years with DCLK1-IN-1, being the most selective and well reported [41-43]. DCLK1-IN-1 is a highly selective DCKL1 inhibitor which has been shown to alter the ATP binding site of DCLK1 without impacting its MAP function [42]. To date it has been efficacious in patient-derived organoid models of PDAC and found to attenuate CRC growth in a syngeneic mouse model without causing significant changes in mouse body weight [42-44]. Use of DCLK-IN-1 in recent years has elucidated not only a metastatic role for DCLK1 in tumorigenesis but also highlighted an immunomodulatory role by regulating the type II immune response within the tumor microenvironment (TME) [45-48]. Overexpression of DCLK1 in PDAC cells engrafted *in vivo* not only accelerated tumor growth but tumors were associated with reduced CD4^+^ and CD8^+^ T cell populations. Furthermore, immunosuppressive M2 polarized macrophages were enriched in DCLK1-overexpressing tumors suggesting DCLK1-mediated formation of an immunosuppressive TME [47]. Importantly, DCLK1-IN-1 restored T cell activity in DCLK1-overexpressing tumors [47]. Analysis of patient data sets in gastric cancer (GC) similarly found DCLK1 expression to correlate with M2 polarized macrophages, suggesting a potential regulatory role in tumor-associated macrophage (TAM) polarization that requires further validation [45]. This immunosuppressive effect may also be mediated by recruitment of myeloid derived suppressor cells (MDSC) with evidence of DCLK1/p-ERK-mediated promotion of C-X-C motif ligand 1 (CXCL1) recruiting MDSCs to the TME in CRC [48]. Recruited MDSCs can then inhibit CD4^+^ and CD8^+^ T cell anti-tumor response, allowing for tumor progression [48]. Alternatively, DCLK1 knockout in CRC tumor cells restores the CD4^+^/CD8^+^ tumor response emphasizing that DCLK1 targeting may not only impede tumor progression and metastasis but improve the adaptive T cell anti-tumor response [48]. This is also supported by data in RCC with use of DCLK-IN-1 found to significantly reduce expression of immune checkpoint ligand PD-L1 in RCC cells [46]. DCLK-IN-1 was also found to restore cisplatin efficacy in resistant ovarian cancer cell lines, further emphasizing DCLK1 targeting as a method for restoring the anti-tumor immune response in a multitude of cancer types [49]. Of note, immunomodulatory effects of DCLK1 may be isoform specific, and considering that expression of the long vs short isoforms has not yet been identified in HNSCC suggests the need for continued investigation into DCLK1 immunomodulatory effects in these patients.

Research investigating the development of invadopodium inhibitors have focused primarily on EGFR inhibitor erlotinib and Src inhibitor dasatanib which have been shown to suppress invadopodia formation and tumor cell invasion in multiple cancer models [50]. In studies by Arnold *et al.*, dasatanib was used to evaluate changes in invadopodia and invasive dynamics, with results indicating DiFiD to be comparable or even more effective in inhibiting invasion [36]. Specifically, dasatanib treatment was found to reduce active MMPs in conditioned media, disrupt EGF stimulated colocalization of DCLK1, KIF16B, and TKS4, as well as colocalization of DCLK1 with KIF16B and RAB40B [36]. Considering these effects to also be seen with DiFiD treatment, suggests DCLK1 inhibition to be a viable target for invadopodia inhibition across cancer.

In conclusion, using an array of molecular experimental techniques, Arnold *et al*., established for the first time that DCLK1 kinase activity mediates early signaling cascades by phosphorylating proteins including EGFR, ERK1/2, Src, and PAK1. Furthermore, the paper identifies a novel mechanism of MMP trafficking to the tip of the invadopodia for matrix degradation, with DCLK1 interaction with KIF16B in invadapodia mediating trafficking and secretion of MMP9-containing vesicles. In conclusion, the evidence that DCLK1 has a causal role in invasion is clear with Arnold *et al.* providing evidence that DCLK1 is a part of large molecular complexes that regulate invadopodia function. However, confirmation of whether MMP-mediated effects are due to DCLK1 kinase activity (i.e. potential phosphorylation of KIF16B or RAB40B) or due to MAP function via DCX domains requires further validation. Understanding whether DCLK1 short or long isoforms mediate HNSCC malignancy and invasion should help to elucidate the regulatory role of DCX domains and whether targeting would be therapeutically relevant for highly invasive cancers like HNSCC. DCLK1 kinase inhibitors such as DiFiD and DCLK-IN-1 have indicated the translatable potential for small molecule targeting inhibiting cellular invasion and mediating restoration of anti-tumor immune responses, emphasizing the potential of these agents in DCLK1 overexpressing cancers. However more data in the realm of *in vivo* work is needed. In aggregate, the evidence of DCLK1 in cancer progression and metastasis is clear and continued investigation into the molecular mechanisms of invasion, isoform specific biological effects, and impact of kinase vs MAP inhibition should help to clarify the translational potential of targeting this protein in oncology patients.
